# Intraductal papilloma in an axillary lymph node of a patient with human immunodeficiency virus: a case report and review of the literature

**DOI:** 10.1186/1752-1947-8-162

**Published:** 2014-05-23

**Authors:** Hannah Cottom, Bhavani Rengabashyam, Philip E Turton, Abeer M Shaaban

**Affiliations:** 1 Department of Histopathology, Leeds Teaching Hospitals NHS Trust, Leeds, UK; 2 Leeds Teaching Hospitals NHS Trust, Leeds, UK; 3 Department of Pathology, University Hospitals Birmingham NHS Foundation Trust, Birmingham, UK

**Keywords:** Axillary lymph node, Ectopic breast tissue, HIV-infection, Intraductal papilloma

## Abstract

**Introduction:**

Inclusions of ectopic breast tissue in axillary lymph nodes are reported very infrequently and typically are only identified microscopically as an incidental finding. Furthermore the development of a benign proliferative lesion in the form of an intraductal papilloma from intranodal ectopic breast tissue is an extremely rare phenomenon with only three previous cases reported. This report describes an unusual and rare case of an intraductal papilloma arising in an axillary lymph node of a patient known to have the human immunodeficiency virus.

**Case presentation:**

A 40-year-old Black African woman underwent excision of an enlarged palpable axillary lymph node. In the preceding 7 years she had received at least six separate surgical excisions to her ipsilateral breast for papillomatosis. The last surgical intervention was performed 1 year prior to presentation with an enlarged axillary lymph node. Histological examination of her axillary lymph node revealed a papillomatous proliferative epithelial lesion within an apparent encompassing duct, resembling a mammary intraductal papilloma. In the surrounding lymphoid tissue small groups of duct-like structures were additionally noted. Immunostaining with a panel of myoepithelial markers in conjunction with oestrogen receptor produced a mixed heterogeneous staining pattern in both the papillomatous lesion and the peripheral duct-like structures. This confirmed the diagnosis of a benign intraductal papilloma within an axillary lymph node, considered to have arisen from ectopic breast tissue.

**Conclusions:**

This case demonstrates that intranodal ectopic breast tissue has the potential to undergo benign proliferative change albeit extremely rarely. Therefore this possibility must be considered to ensure the correct diagnosis is made. In addition, to the best of our knowledge, this is the first case report which has described recurrent intraductal papillomas and the subsequent development of an intraductal papilloma within an ipsilateral axillary lymph node, in a patient who is human immunodeficiency virus positive. There is minimal literature investigating the specific types of breast pathologies experienced by patients infected with human immunodeficiency virus and it remains unexplored as to whether human immunodeficiency virus may lead to proliferative papillomatous epithelial changes. This report considers the role of the human papillomavirus and recommends that further investigatory studies are required.

## Introduction

Intraductal papillomas (IDPs) are discrete benign tumours arising in mammary ducts. They comprise a proliferation of epithelial and myoepithelial cells which are supported by fibrovascular cores, producing a characteristic arborescent architecture within the duct lumen [1]. Ectopic breast tissue (EBT) usually develops along the embryological milk line with recognition that benign EBT may occur in the axillary nodes. EBT predominantly comprises single or small groups of glands and ducts which exhibit a varied morphological appearance, although the majority are composed of luminal epithelial cells and basal cells demonstrating myoepithelial differentiation [2]. Axillary intranodal ectopic breast inclusions are however an infrequent finding and typically are only identified microscopically as an incidental finding [2-4]. The development of a benign proliferative lesion such as an IDP from intranodal EBT is an extremely rare phenomenon. A review of the literature has revealed only three reports of an IDP arising within an axillary lymph node [4-6]. In this report we describe a further unusual case of an IDP arising in an axillary lymph node of a patient known to have the human immunodeficiency virus (HIV) and a previous history of recurrent IDPs of the ipsilateral breast.

## Case presentation

Our patient was a 40-year-old Black African woman with a medical history of HIV infection and mild depression. She was a non-smoker and had no known drug allergies. Her medication included venlafaxine, and reverse transcriptase inhibitors efavirenz (non-nucleoside) and lamivudine (nucleoside). She had originally attended 4 years previously with left nipple discharge associated with pain and discomfort of her left breast. Ultrasound imaging with core biopsies revealed multiple benign IDPs in her left breast and she underwent a Hadfield’s procedure (radical subareolar duct excision). Another Hadfield’s procedure was performed in the following year (2007) after recurrence of the benign IDPs. In 2010 she underwent a left subcutaneous mastectomy with implant reconstruction following development of a further IDP in her left breast. This form of surgical treatment was decided most appropriate due to the extent of the IDP, difficulty of radiological follow-up and to exclude atypia and risk of subsequent malignancy. In the mastectomy specimen a single IDP was present measuring approximately 38×22mm in maximum dimension. The IDP contained several suspicious foci of solid atypical proliferations, which after immunohistochemical staining were regarded as atypical ductal hyperplasia. The degree of atypia was not considered to amount to ductal carcinoma *in situ* (DCIS) and there was no evidence of invasive neoplasia. No axillary lymphadenopathy was noted at the time of the mastectomy. *In situ* hybridisation for the detection of human papillomavirus (HPV) was performed on two separate IDPs (in 2008 and the IDP in the mastectomy specimen in 2010). In both cases no HPV was detected with the probe set used (HPV 1, 2, 6, 11, 16, 18, 31 and 33). During follow up, 2 years after her left mastectomy (in 2012), she reported a small lump in her left breast. Ultrasound imaging revealed a new 9mm well-defined hypoechoic mass. This was shown on core biopsy to be a further IDP. Diagnostic excision of the lesion confirmed a benign IDP with hyperplasia, apocrine metaplasia and no evidence of atypia.

In 2013 she developed an enlarged palpable mass in her left axilla.

### Diagnostic focus and assessment

**Figure 1:**
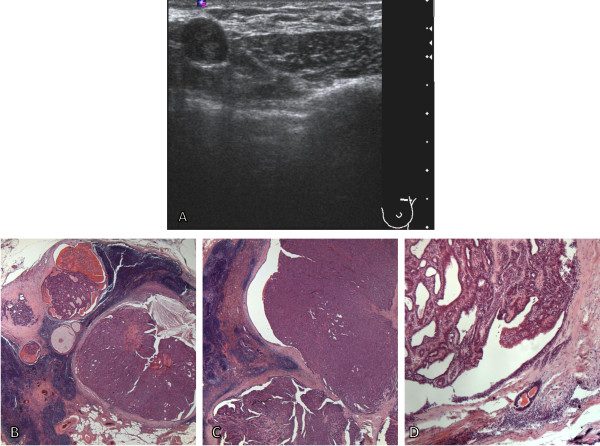
**Ultrasound imaging and photomicroscopy of the axillary intraductal papilloma. A** Ultrasound imaging of the axillary lymph node. **B** and **C**: Histological appearance of the intraductal papilloma in the left axillary lymph node, magnification ×10. **D**: Illustration of duct-like epithelial structures in the adjacent lymphoid tissue considered to represent ectopic breast tissue, magnification ×20. **E-G**: Immunohistochemical staining profile of the intraductal papilloma with mixed positivity to cytokeratin 5 **(E)**, smooth muscle myosin **(F)** and p63 **(G)**, magnification ×20.

On ultrasound imaging the axillary mass corresponded to an abnormally enlarged lymph node with eccentric cortical thickening and loss of medullary fat (Figure 1A). No changes or abnormalities were detected in her left breast. Left axillary ultrasound-guided core biopsies of the lymph node revealed part of an IDP. As a result of this extremely unusual finding a diagnostic excision of the enlarged left axillary lymph node was performed. The lymph node measured 20mm in maximum dimension. The histology of the lymph node showed a reasonably well-circumscribed papillomatous proliferative epithelial lesion within an apparent encompassing duct. The lesion was confined to the node and comprised prominent tightly packed papillary fronds lined by a bilayer of luminal epithelial cells and an outer layer of basal cells supported by fibrovascular cores (Figures 1B and 1C); identical to an IDP of the breast. Cholesterol crystals, dense collections of macrophages and reactive stromatolites were also noted in part, signifying a long standing lesion and the latter indicative of previous core biopsy. No necrosis or significant atypia was evident and few mitoses were identified. At the periphery, benign lymphoid tissue was seen confirming the intranodal location of the lesion. In this surrounding lymphoid tissue small groups of duct-like structures were additionally noted (Figure 1D). In view of the curious finding of an IDP within an axillary lymph node immunohistochemical staining was performed for confirmatory characterisation of the lesion and exclusion of malignancy. The lesion and encompassing duct demonstrated mixed cytokeratin (CK) 5 and 14, p63 and smooth muscle myosin positivity (Figures 1E-G) with heterogeneous oestrogen receptor (ER) expression, indicating a benign lesion. A similar staining pattern was also displayed in the duct-like epithelial structures thereby determining their origin as breast. The histological appearances and immunohistochemical staining profile therefore substantiated a benign IDP within her left axillary lymph node, which was considered to have arisen from EBT.

She experienced an uneventful postoperative period and remains well at follow up appointments. Her recent annual contralateral mammogram (performed 1-month post-lymph node excision) revealed no significant interval change in her right breast when compared with previous mammograms, and no palpable axillary lymph nodes were identified.

## Conclusions

It is estimated that EBT is found in approximately 1% of the population [7]; however, its presence in axillary lymph nodes is uncommon and when it does occur it is frequently an incidental microscopic finding [2-4]. The aetiology of intranodal EBT remains unclear. It has been postulated that it may result from embryological maldevelopment leading to entrapment of mammary epithelial cells within nodal tissue or as a consequence of cell embolism following breast surgery [4,8-10]. Table 1 summarises the three previously reported cases of IDP arising within an axillary lymph node and our case.

**Table 1:** Summary of previous reports and current case report of an intraductal papilloma arising within an axillary lymph node

**Author, Reference number (year)**	**Patient age (years)**	**Previous clinical history**	**Presenting features of intranodal IDP**	**Size (mm)**	**Suspected aetiopathogenesis**
Ichihara *et al*. [4] (2008)	47	5 ipsilateral IDPs in the preceding 5 years	Incidental discovery during sentinel lymph node biopsy	6	Postulation of mammary epithelial cell dissemination following multiple surgical manipulations to ipsilateral breast OR from EBP
Dzodic *et al*. [5] (2010)	34	Single ipsilateral IDP 10 years previously	Presentation as palpable axillary lymph node	11	Favoured evolvement from intranodal EBT as duct-like structures identified in the background lymphoid tissue
McDiwitt *et al*. [6] (1968)	Not stated	Not stated	Not stated	Not stated	Description of case only
Current case (2014)	40	>6 ipsilateral IDPs in the preceding 7 years	Presentation as palpable axillary lymph node	20	Favoured evolvement from intranodal EBT as duct-like structures identified in the background lymphoid tissue

Abbreviations: EBT, ectopic breast tissue; IDP, intraductal papilloma.

The behaviour and evolution of EBT is unpredictable and particularly unexplored in relation to axillary lymph nodes owing to its rarity. Several studies have demonstrated EBT to exhibit squamous and apocrine metaplasia, cystic change and epithelial hyperplasia [3,4,8-10]. These findings would suggest that EBT is susceptible to similar hormonal influences and changes as native mammary tissue. Accordingly, it is feasible for benign proliferative lesions such as IDPs to develop from such EBT, albeit exceptionally rare within the axillary lymph nodes. It is therefore also conceivable that malignant transformation is possible. This is incredibly rare with only two case reports identified in the literature [11,12]. Jaffer *et al*. (2008) in their case report described transformation of an IDP within an axillary lymph node into an intraductal carcinoma. Their patient had a previous history of an ipsilateral lumpectomy for an IDP which displayed atypical ductal hyperplasia and focal DCIS [11]. The other case report documented a case of papillary carcinoma arising in an axillary lymph node also considered to have originated from EBT [12]. Therefore an extremely important ramification of intranodal EBTs is to ensure that they are not misinterpreted as either metastatic carcinoma or a primary malignancy. This may be of particular diagnostic difficulty if there has been proliferative change. Nevertheless pathologists and clinicians must be mindful of the possible occurrence of EBT in axillary lymph nodes and the very infrequent proliferative changes that it may display. In the current case, cystic change associated with cholesterol deposition indicated a long standing lesion. Focal disruption to the IDP within the axillary lymph node was also noted with small epithelial islands seen to extend beyond the encompassing duct. This was however found in association with inflammation and reactive changes consistent with a previous core biopsy site. It was therefore considered artefactual in nature representing epithelial displacement within. In addition, the recognition and demonstration of myoepithelial cells and the heterogeneous ER and CK5 immunohistochemical staining confirmed the lesion as benign.

In our case report the patient was known to be HIV positive and had undergone to this date at least six separate surgical excisions to her left breast for papillomatosis. This included two Hadfield’s and an eventual left mastectomy and breast reconstruction. There is minimal literature investigating the specific types of breast pathologies experienced by patients infected with HIV. To the best of our knowledge this is the first case report which has described recurrent IDPs and the subsequent development of an IDP within an ipsilateral axillary lymph node, in a patient who is HIV positive. It is contemplated that HIV may affect the glandular elements of breast tissue in seropositive patients [13], although it remains unexplored as to whether this may lead to proliferative papillomatous epithelial changes. It has been demonstrated that in patients with HIV-1 disease, such as in our patient, there is greater infection with HPV in multiple locations including the nipple [14]. HPV is widely recognised as the cause of papillomas in multiple sites and therefore could have a role to play in the aetiopathogenesis of IDPs [15]. Currently though there is limited information on this relationship and certainly in our case no HPV was detected. Further studies investigating the potential role of HPV in IDPs and the effects of HIV on the breast and EBT are required.

## Consent

Written informed consent was obtained from the patient for publication of this case report and accompanying images. A copy of the written consent is available for review by the Editor-in-Chief of this journal.

## Abbreviations

CK: Cytokeratin; DCIS: Ductal carcinoma *in situ*; EBT: Ectopic breast tissue; ER: Oestrogen receptor; HIV: Human immunodeficiency virus; HPV: Human papillomavirus; IDP: Intraductal papilloma.

## Competing interests

The authors declare that they have no competing interests.

## Authors’ contributions

HC was the major contributor in writing the manuscript. AS directed and assisted with writing the manuscript and performed the histological examination of the case. EPT assisted with writing the manuscript and provided details on the surgical management of the patient. BR performed the radiological examination of the case. All authors read and approved the final manuscript.
